# Chronic Kidney Disease: The Complex History of the Organization of Long-Term Care and Bioethics. Why Now, More Than Ever, Action is Needed

**DOI:** 10.3390/ijerph16050785

**Published:** 2019-03-04

**Authors:** Elisabetta Versino, Giorgina Barbara Piccoli

**Affiliations:** 1 Department of Clinical and Biological Sciences, University of Torino, 10124 Torino, Italy; 2 Nephrology, Centre Hospitalier Le Mans, 72000 Le Mans, France

**Keywords:** CKD, dialysis, kidney transplantation, mortality, morbidity and costs of care

## Abstract

Chronic kidney disease (CKD) has been redefined in the new millennium as any alteration of kidney morphology, function, blood, or urine composition lasting for at least 3 months. This broad definition also encompasses diseases or conditions that are associated with normal kidney function, such as a kidney scarring from an acute pyelonephritis episode or a single kidney, as a result of kidney donation. CKD is a relevant public health problem. According to the 2015 Global Burden of Disease Study, it was the 12th leading cause of death, leading to 1.1 million deaths, worldwide, each year. The role of CKD as a cause of death is evident where renal replacement therapy (RRT) is not available, however, its role in increasing death risk is not easily calculated. RRT consumes about 3–5% of the global healthcare budget where dialysis is available without restrictions. While the prevalence of CKD is increasing overall as lifespans extend, being linked to diabetes, hypertension, obesity, and atherosclerosis, CKD is at least partly preventable and its effects may be at least partly counterbalanced by early and appropriate care. We will welcome papers on all aspects of CKD, including organization, cost, and models of care. Papers from developing countries will be particularly welcomed.

“*In public health, we can’t do anything without surveillance. That’s where public health begins*.”

—*David Satcher, MD, PhD, U.S. Surgeon General, in 1998–2002*

Chronic kidney disease (CKD) is a major public health problem [1]. The 2015 Global Burden of Disease Study estimated that, in 2015, 1.2 million people died from CKD [2]. This umbrella term (which at the beginning of the new millennium replaced the previously used label, renal insufficiency) gathers all persistent alterations (those lasting at least three months) in morphology, urinary or blood composition, or any reduction in the glomerular filtration rate below 60 mL/min [3].

The definition of CKD emphasizes the importance of the early phases of kidney damage, in which, due to the great functional reserve of kidney tissue, more than 50% of the kidney tissue has to be damaged before producing a rise in serum creatinine and a reduction in kidney function. In fact, this is the reason for the diffusion of living-donor kidney transplantation, a treatment aimed at restoring the health of a patient with end-stage kidney disease without producing detrimental effects on the health of the donor [4,5].

The relationship between kidney tissue, kidney function, and health is complex. On one hand, as in the case of kidney donation, reduction of kidney tissue can be fully compensated without any effect on kidney function in standard situations. On the other hand, any reduction in kidney tissue, even in the presence of normal kidney function, can have a negative effect on conditions, for example during pregnancy, in which renal function is subject to stress [6]. It is only in the new millennium that the prevalence of CKD, the Cinderella of chronic diseases, has come to be fully appreciated. We now realize that in western countries CKD is present in 5–12% of the overall population, depending on the way its presence is detected and the features of the population. Its prevalence is therefore between that of diabetes mellitus and hypertension, much better known non-transmissible chronic diseases [1]. There seems to have been no decrease in the prevalence of CKD, which is not surprising given that it is more commonly found in older people and it is linked with the major non-transmissible diseases (hypertension, obesity, diabetes, and atherosclerosis). While in western countries the rise in the prevalence of CKD is generally in line with the ageing of the overall population, a sharp rise has been observed in emerging countries. This is more closely linked to a rise in diabetes and obesity, the price paid for adopting a “westernized” way of life, than to increased life expectancy [7,8].

The relationship between kidney disease mortality and morbidity is complex. Where renal replacement therapy is available a loss of over 90% of kidney function is compatible with decades of survival on this therapy [9]. On the other hand, the presence of any degree of reduction in kidney function is associated with an increased risk of death, directly proportional to reduction in kidney function [10]. Although this complex relationship makes the estimation of the role of kidney disease as a cause of death difficult to quantify within these limits, CKD is considered the 12th cause of death worldwide.

Conversely, the economic burden of end-stage kidney disease [ESRD] is more easily quantified. Renal replacement therapy [RRT], in the settings where it is available without restriction, consumes between 2% and 5% of the entire health care budget for a cohort that is approximately 1:1000 of the overall population. While kidney transplantation is the elective treatment for ESRD, with better overall survival results and lower costs, very old patients and those with multiple comorbidities are not considered eligible [11]. In most European countries the median age at the start of dialysis is about 75 and, while the conventional age limit for transplantation is now usually set at about 80, the prevalence of contraindications increases with age. As a result, the prevalence of kidney transplant candidates ranges from 40% to 70% according to the setting and the system of care [12].

Dialysis is extremely expensive and while there is no single way to calculate costs, given significant differences in policies, health care systems, and reimbursement policies, in Europe one year of dialysis is calculated to cost between 50,000 and 100,000 euros [11].

Dialysis is not available for all patients who need it. This is a tremendous health care problem affecting not only chronic patients but also patients with acute kidney diseases that require dialysis to allow kidney healing [13]. In 2010, an estimated 2.3–7.1 million people with end-stage kidney disease died without access to chronic dialysis [14]. The courageous program of the International Society of Nephrology, entitled ‘0 by 25’, is addressed to ensuring the availability of dialysis for all patients with an acute kidney injury [AKI], thus avoiding over 1.5 million deaths worldwide every year [13] (Figure 1).

As is so often the case in this difficult world, the need is greatest in low-income countries, where, for instance, AKI is the main cause of death during and after pregnancy [15].

On account of the high prevalence of CKD, the high cost, and unequal availability of treatment, ethical issues are an integral part of the history of nephrology and its present development. The first ethical committee known in the western world was set up in Seattle in the early 1960s to share the burden involved in deciding which patients should be put on dialysis, or “who should live”. In fact, in the early days of chronic dialysis the availability of an “artificial kidney” was so limited that choosing candidates was felt to require shared responsibility and the “God Committee” was therefore the first tragic example of the impact of economic and social factors on clinical choices (Figure 2) [16].

While the involvement of politicians and the media led to “open access”, i.e., widening the availability of dialysis in North America and Europe, this tragic choice still confronts physicians elsewhere in the world in those regions which are, today, the most populated, poorest, and have highest incidence of kidney disease.

From a public health perspective, the following are the main critical points:

- How to estimate the true prevalence and economic burden of CKD
- How to implement efficient surveillance systems
- How to test new models of prevention and health care organization
- How to evaluate their effectiveness
- How to ensure equity in access to preventive medicine and care
- How to ensure the ecological sustainability of kidney treatments

The concept of public health surveillance [PHS] is linked to all these critical points, as PHS is “the systematic and continuous collection, analysis, and interpretation of data, closely integrated with the timely and coherent dissemination of the results and assessment to those who have the right to know so that action can be taken” [17]. PHS is a complex system, collecting information from various sources and using it as the basis of decision making. Unfortunately, given a paucity of epidemiological data, lack of awareness, and limited access to testing, the actual burden of kidney disease is probably underestimated. PHS could contribute to increasing equity by underlining inequality, not only between social strata but also between areas and nations [18].

The global burden of kidney disease is growing, driven by complex interactions, and treatment is fraught with environmental and socioeconomic disparities. Although CKD is costly, it is also at least partly preventable and adverse outcomes can be delayed, often by employing low cost treatments [19]. We need universal health coverage to ensure effective screening, prevention, and early treatment of CKD. Involvement of all relevant stakeholders and finding alternative financing strategies is necessary to promote equal access to care [20].

There is need for action and action starts from awareness. This is the reason why we feel that this special issue will be welcomed as a means to strengthen the links between clinical nephrologists, economists, and policy makers.

## Figures and Tables

**Figure 1:**
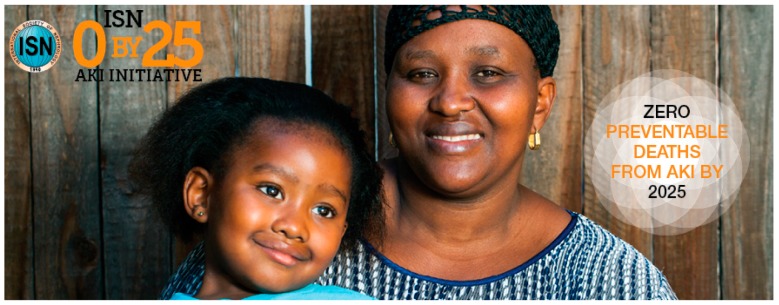
One of the icons of the “O by 25” campaign of the International Society of Nephrology; Available online: https://www.theisn.org/all-articles/616-0by25 (accessed on 25 February 2019).

**Figure 2:**
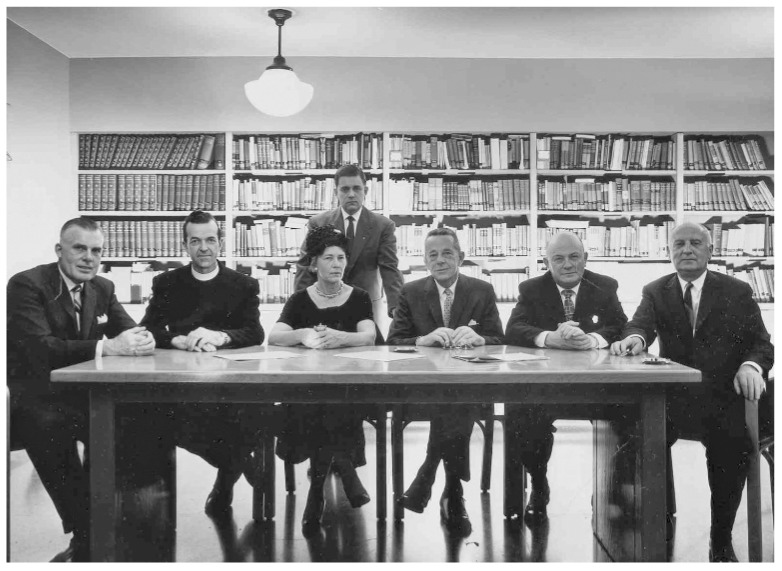
The “God Committee” selecting patients for dialysis. West of the Rotunda—Difficult choices. Available online: http://danschmidtforsenate.com/blog/?p=291 (accessed on 23 January 2019).
